# 4346 Potential Sudden Unexpected Death in Epilepsy (SUDEP) Biomarkers in Human Induced Pluripotent Stem Cell-Derived Cardiomyocytes with DEPDC5 Loss-of-Function

**DOI:** 10.1017/cts.2020.311

**Published:** 2020-07-29

**Authors:** Yanting Zhao, Helen Zhang, Jack M. Parent, Lori L. Isom

**Affiliations:** 1 University of Michigan School of Medicine; 2 University of Michigan; 3 Department of Pharmacology, University of Michigan

## Abstract

OBJECTIVES/GOALS: Sudden Unexpected Death in Epilepsy (SUDEP) is a leading cause of death in epilepsy patients. This study aims to determine whether cardiac mechanisms contribute to SUDEP in epilepsy patients with variants in *DEPDC5*, a gene encoding a member of the mTOR GATOR complex, to identify SUDEP biomarkers. METHODS/STUDY POPULATION: SUDEP has been reported in 10% of epilepsy patients with *DEPDC5* loss-of-function variants. We used human induced pluripotent stem cell-derived cardiomyocytes (iPSC-CMs) to measure changes in cellular excitability that are known to be substrates for cardiac arrhythmias. CRISPR-derived isogenic *DEPDC5* iPSC-CMs and *DEPDC5* patient-derived iPSC-CMs were used in this study. Whole-cell patch-clamp was used to measure voltage-gated sodium current (*I*_Na_) and calcium current (*I*>_Ca_) in single iPSC-CMs in voltage-clamp mode; and to measure action potentials (APs) in 3-dimentional iPSC-CM-derived micro-tissues in current-clamp mode. RESULTS/ANTICIPATED RESULTS: CRISPR generated heterozygous deletion of 1 base-pair in the first coding exon of *DEPDC5* gene, resulting in a premature stop codon, simulated the variants identified in *DEPDC5* epilepsy patients. In CRISPR generated heterozygous*DEPDC5* iPSC-CMs, whole-cell voltage-clamp recordings revealed that *I*_Na_ was increased and *I*_Ca_ was reduced compared with isogenic control iPSC-CMs. Whole-cell current-clamp recordings revealed that AP duration at 80% and 90% of repolarization, APD_80_ and APD_90_, respectively, were prolonged compared to isogenic control iPSC-CMs. Similar measurements will be performed for iPSC-CMs derived from *DEPDC5* patients. DISCUSSION/SIGNIFICANCE OF IMPACT: This study shows that epilepsy patients with non-ion channel gene variants in *DEPDC5* have altered CM excitability, which may serve as a substrate for cardiac arrhythmias in *DEPDC5* patients. Importantly, this work may allow us to identify biomarkers for SUDEP risk in these patients in the future. CONFLICT OF INTEREST DESCRIPTION: L.L.I. is the recipient of a collaborative research grant from Stoke Therapeutics.

